# Nanoscale Particulate Matter from Urban Traffic Rapidly Induces Oxidative Stress and Inflammation in Olfactory Epithelium with Concomitant Effects on Brain

**DOI:** 10.1289/EHP134

**Published:** 2016-05-17

**Authors:** Hank Cheng, Arian Saffari, Constantinos Sioutas, Henry J. Forman, Todd E. Morgan, Caleb E. Finch

**Affiliations:** 1 Leonard Davis School of Gerontology,; 2 USC Dornsife College,; 3 Viterbi School of Engineering, University of Southern California, Los Angeles, California, USA

## Abstract

**Background:**

Rodent models for urban air pollution show consistent induction of inflammatory responses in major brain regions. However, the initial impact of air pollution particulate material on olfactory gateways has not been reported.

**Objective:**

We evaluated the olfactory neuroepithelium (OE) and brain regional responses to a nanosized subfraction of urban traffic ultrafine particulate matter (nPM, < 200 nm) in vivo, ex vivo, and in vitro.

**Methods:**

Adult mice were exposed to reaerosolized nPM for 5, 20, and 45 cumulative hours over 3 weeks. The OE, the olfactory bulb (OB), the cerebral cortex, and the cerebellum were analyzed for oxidative stress and inflammatory responses. Acute responses of the OE to liquid nPM suspensions were studied with ex vivo and primary OE cultures.

**Results:**

After exposure to nPM, the OE and OB had rapid increases of 4-hydroxy-2-nonenal (4-HNE) and 3-nitrotyrosine (3-NT) protein adducts, whereas the cerebral cortex and cerebellum did not respond at any time. All brain regions showed increased levels of tumor necrosis factor-α (TNFα) protein by 45 hr, with earlier induction of TNFα mRNA in OE and OB. These responses corresponded to in vitro OE and mixed glial responses, with rapid induction of nitrite and inducible nitric oxide synthase (iNOS), followed by induction of TNFα.

**Conclusions:**

These findings show the differential time course of oxidative stress and inflammatory responses to nPM between the OE and the brain. Slow cumulative transport of inhaled nPM into the brain may contribute to delayed responses of proximal and distal brain regions, with potential input from systemic factors.

**Citation:**

Cheng H, Saffari A, Sioutas C, Forman HJ, Morgan TE, Finch CE. 2016. Nanoscale particulate matter from urban traffic rapidly induces oxidative stress and inflammation in olfactory epithelium with concomitant effects on brain. Environ Health Perspect 124:1537–1546; http://dx.doi.org/10.1289/EHP134

## Introduction

The brain has emerged as a target of air pollution. Population-based studies have shown that cognitive impairments increase in proportion to levels of PM_2.5_ (Ailshire and Crimmins 2014; Gatto et al. 2014; Tonne et al. 2014) and ozone; these impairments approximate 3–5 years of accelerated cognitive loss (Chen and Schwartz 2009). Correspondingly, white matter loss was increased by 1% per 3 μg/m^3^ PM_2.5_ in a magnetic resonance imaging (MRI) analysis of elderly women of the Women’s Health Initiative Memory Study cohort (Chen et al. 2015). Cortical white matter volume changes and inflammation were also reported in a small sample of postmortem children from a highly polluted Mexican City (Calderón-Garcidueñas et al. 2011). Rodent models further document inflammatory responses of the cortex, olfactory bulb (OB), and midbrain to relatively short-term exposure to automotive-derived air particulate matter (Block et al. 2012; Campbell et al. 2005; Levesque et al. 2011b; Morgan et al. 2011). Notably, tumor necrosis factor-α (TNFα) induced by nanosized particulate matter (nPM) can impair neurite outgrowth (Cheng et al. 2016).

In the present sudy, we focus on the ultrafine class of PM (i.e., particles with diameter < 200 nm) to extend our prior studies (Cheng et al. 2016; Morgan et al. 2011). Additionally, ultrafine PM has higher *in vivo* and *in vitro* toxicity than larger PM derived from combustion engines (Gillespie et al. 2013; Li et al. 2003). Moreover, in inhalation studies, nanosized particles could physically translocate to the OB and brain via the axons of olfactory sensory neurons in the olfactory neuroepithelium (OE), which project directly to synapses in the OB glomerulus (Elder et al. 2006; Oberdörster et al. 2004). These observations are supported by nasal instillation studies of ultrafine particles, which show translocation to the OB and induce TNFα and macrophage inflammatory protein (MIP)-1α in the OB (Win-Shwe et al. 2006). *In vivo* and *in vitro*, nPM induced interleukin (IL)-1α, IL-6, and TNFα, with glial responses (CD68, GFAP) (Cheng et al. 2016; Morgan et al. 2011). Similarly, nanoscale diesel exhaust (DE) induced TNFα, IL-6, and MIP-1α in olfactory bulb (OB) and post-olfactory brain regions (Levesque et al. 2011b). Thus, the OE may be an important gateway for the impact of ultrafine PM on the central nervous system.

Although there are well-documented OE responses to ozone (Wagner et al. 2002; Ong et al. 2016), little is known about acute OE responses to acute air pollution PM in rodent models. Because the OE is the first neuronal contact of inhaled PM and because OE neuron dendrites regress with acute *in vitro* exposure to nPM (Cheng et al. 2016), we hypothesized that OE responses would be rapid and would precede brain responses. We therefore defined the time-course response of OE, OB, cerebral cortex, and cerebellum to nPM *in vivo* for oxidative stress [4-hydroxy-2-nonenal (4-HNE) and 3-nitrotyrosine (3-NT)] (Butterfield et al. 2011) and inflammatory responses (TNFα and microglia) (Kraft and Harry 2011). Furthermore, we introduce an *ex vivo* model of the OE for studying acute responses to nPM. These exposure paradigms used a chemically defined nanoscale subfraction that we designated as nPM to distinguish it from the total ultrafine PM_0.2 μm_ class (Morgan et al. 2011).

## Material and Methods

### nPM Collection and Extraction

Nanoscale particulate matter (nPM, < 0.2 μm in diameter) was collected on Teflon filters by a high-volume ultrafine particle (HVUP) sampler (Misra et al. 2002) at a 400-L/min flow, ~150 m downwind of the I-110 Freeway in central Los Angeles, California. nPM collected at this location between August and September 2012 represents urban ultrafine particles, predominantly originating from vehicular combustion emissions in addition to other, less-substantial sources such as submicron road dust (Hasheminassab et al. 2013; Saffari et al. 2013). The composition of the collected nPM samples was similar to that of samples collected in prior studies (Morgan et al. 2011). Filter-trapped, dried nPM was eluted by sonication into deionized water. nPM suspensions (150 μg/mL) were tested for sterility (no microbial growth in nutrient media) and stored at –20°C. nPM slurries were endotoxin-free as assayed by Limulus assay (Davis et al. 2013). Fresh, sterile filters were sham extracted to serve as controls for nPM extracts. The slurries were then either reaerosolized for animal exposure or used for treating cell cultures.

### Animals and Ethics Statement

*In vivo* and *ex vivo* studies used 3-month-old adult C57BL/6J male mice (average body weight: 27 g; *n* = 6/treatment/time, 36 total) purchased from Jackson Laboratories. Protocols were approved by the University of Southern California (USC) Institutional Animal Care and Use Committee (IACUC); animals were maintained following National Institutes of Health (NIH) guidelines. Mice were fed Purina Lab Chow and sterile water *ad libitum* and were housed in standard animal housing facilities operated by the USC Department of Animal Resources. Mice were housed in groups of 4 or 5 at 21–22°C with 30% humidity and were housed in cages with standard bedding and nesting material that allowed for ample free movement. The light cycle time range was 0600–1800 hours. For tissue collection, starting at 0900 hours, animals were euthanized by standard isoflurane anesthesia followed by cervical dislocation, and were then perfused with saline. Organs were immediately frozen on dry ice after collection for storage. Animal procedures were performed at 21–23°C.

*In vitro* studies with primary cultures used pups from Sprague Dawley rats (received on pregnancy day 14) from Harlan Labs. Three pregnant rats were received for three experimental replicates and were housed under standard conditions (Morgan et al. 2011). Pups were housed with the mothers prior to use at postnatal day 3 (P3).

### Exposure Conditions

Mice were randomly assigned to groups and were then exposed to nPM that was reaerosolized by a HOPE nebulizer (B&B Medical Technologies) (Wang et al. 2013) at 343 μg/m^3^ for 5 hr (1000–1500 hours)/day, 3 day (MWF)/week for cumulative 5, 20, or 45 hr (see Figure S1). The nPM slurry was dehydrated and charge-neutralized in the nebulizer chambers before mouse exposure. Particle number concentration of the inlet aerosol was monitored during exposures by a condensation particle counter (CPC; TSI Inc.) (Morgan et al. 2011; Wang et al. 2013). For exposure, mice were transferred from their home cages into sealed exposure chambers that allowed adequate ventilation; individuals were separated to minimize aggression. Exposed mice remained healthy and did not incur changes in body weight or core temperature compared with controls measured before euthanasia (not shown).

### Cell Culture

**Figure 1:**
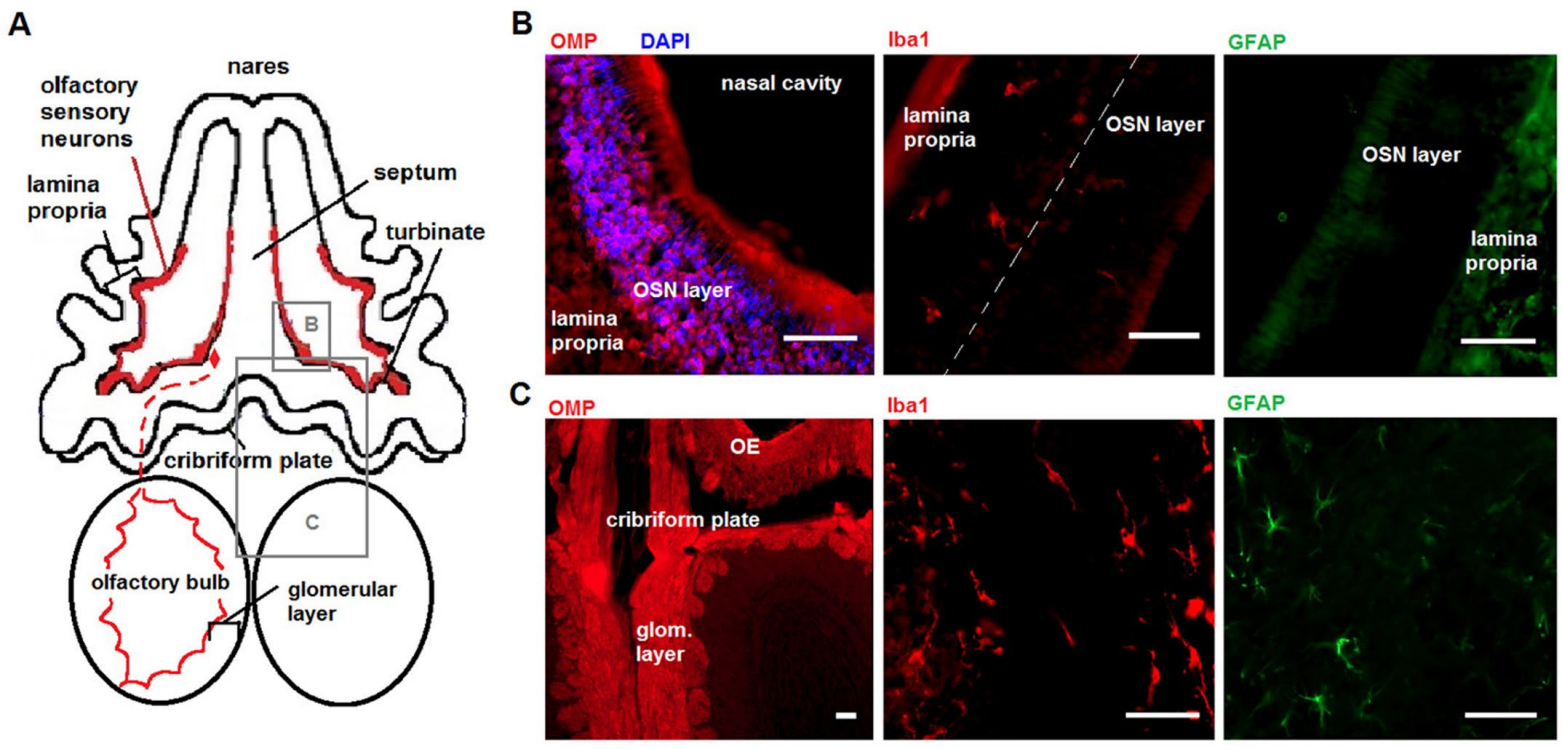
Model of olfactory gateway to brain. Source: Part (*A*) reproduced from Cheng et al. (2016), *Journal of Neuroinflammation*, under terms of the Creative Commons Attribution 4.0 International License (http://creativecommons.org/licenses/by/4.0/). Some changes have been made to this figure. (*A*) Schema, dorsal transverse plane, of the olfactory epithelium (OE) and olfactory bulb (OB). The outlined boxes identify displayed regions for OE and OB; the red dashed line represents an olfactory sensory neuron (OSN) and olfactory axon projection to the OB. (*B*) Olfactory epithelium lining the ethmoturbinate space contains OSNs and Iba1-positive macrophages; Glial fibrillary acidic protein (GFAP) shows diffuse staining in the lamina propria but was not detected above background in the OSN layer. Nuclei were identified with 4′,6-diamidino-2-phenylindole (DAPI). (*C*) The OB contains OSN projections in the glomerular layer (glom. layer), astrocytes, and microglia. Olfactory marker protein (OMP) staining is shown at lower magnification for clarity. *Scale bar* *=* 50 μm.

For *ex vivo* OE organ cultures, adult mice were cardiac perfused with phosphate-buffered saline (PBS; pH 7.4), and the nasal mucosa was delaminated as paired tissue ribbons (10 mg wet weight per mouse; two mice pooled per sample). Here, the OE is designated as the nasal mucosa lining the nasoturbinates and ethmoturbinates (Figure 1A). OEs were rinsed in PBS before incubation for 2 hr with 150 μL of nPM (12 μg/mL) diluted in artificial cerebral spinal fluid (aCSF: 119 mM NaCl, 26.2 mM NaHCO_3_, 2.5 mM KCl, 1 mM NaH_2_PO_4_, 1.3 mM MgCl_2_, 10 mM glucose). Conditioned media (CM) were collected, centrifuged at 10,000 × *g* for 5 min, and analyzed. Tissues were processed to obtain RNA.

*In vitro* primary cell culture studies were originated from OE and mixed glia from cerebral cortex of P3 rats. The entire litter (both sexes) was used for primary cultures (~10 pups). For cell cultures, OEs were dissociated via trituration and filtering using a 70-μm cell strainer. Cells were grown for 2 weeks in 6-well plates until reaching 95% confluence; then, nPM was added to the cultures to perform a nitrite time course (Griess assay). At confluence, OE primary cell cultures contained mainly spindle-shaped cells (lacking GFAP, Iba1, and NeuN); a minority of cells (< 5%) expressed GFAP, Iba1, or NeuN (not shown).

For cerebral cortex mixed glia, cultures were grown for 2.5 weeks and consisted of a 3:1 ratio of astrocytes:microglia; these cultures were treated with trypsin to produce secondary cultures in 6-well plates. Secondary cultures were plated at approximately 1,000,000 cells/well. All primary cultures (OE, mixed glia) were grown in Dulbecco’s modified Eagle’s medium/Ham’s F12 50/50 Mix (DMEM F12 50/50) supplemented with 10% fetal bovine serum (FBS), 1% penicillin/streptomycin (pen/strep) and 1% L-glutamine in a humidified incubator (37°C/5% CO_2_) (Rozovsky et al. 1998). To treat the cells, nPM (12 μg/mL) was diluted in DMEM supplemented with 15 mM HEPES, 1% sodium pyruvate, 0.24% bovine serum albumin (BSA), 1% pen/strep, and 1% L-glutamine and applied onto cells for 1, 6, 12, and 24 hr. Cell culture experiments were repeated three times.

### Nitrite Assay

CM were assayed for nitrite with the Griess reagent (Ignarro et al. 1993) using NaNO_2_ as a standard and untreated media as a blank control. Assays were performed using 96-well plates with 50 μL of CM in duplicate and 50 μL Griess reagent for 10 min in the dark at 21–23°C before reading at 550 nm with a SpectraMax M2 microplate reader (Molecular Devices).

### Western Blots

Brain regions (OE, OB, cerebral cortex, cerebellum) were homogenized by a motor-driven pestle on ice in 1× RIPA buffer (Millipore) supplemented with 1 mM phenylmethane sulfonyl fluoride (PMSF), 1 mM Na_3_VO_2_, 10 mM NaF, phosphatase inhibitor cocktails (Sigma), and Roche Complete Mini EDTA-free Protease Inhibitor Cocktail Tablet (Roche). Homogenates were centrifuged at 10,000 × *g* for 10 min at 4°C; the supernatants were analyzed by performing Western blotting using 20 μg of total protein on Novex NuPAGE 4–12% Bis-Tris protein gels (Thermo Scientific). Membranes were washed with PBS with 0.05% Tween-20 (PBST), then blocked with 5% milk/PBST or 5% BSA/PBST for 1 hr at 21–23°C, followed by overnight incubation with primary antibodies at 4°C: anti-TNFα (1:250, mouse; R&D Systems), anti-3-nitrotyrosine (1:1,000, rabbit; Millipore), anti-4-hydroxynonenal (1:250, mouse; R&D Systems), anti-olfactory marker protein (OMP) (1:400, goat; Santa Cruz), anti-cleaved caspase 3 (1:1,000, rabbit; Cell Signaling), anti-caspase 3 (1:1,000, rabbit; Cell Signaling), anti-actin (1:10,000, mouse; Sigma). Horseradish peroxidase (HRP; 1:10,000, goat; Jackson)-enhanced chemiluminescence was detected using West Pico Chemiluminescent Substrate (Thermo Scientific). The density of the bands was assessed using ImageJ (Abràmoff et al. 2004).

### Immunohistochemistry

Following nPM treatment and euthanasia, the animals’ heads were de-skinned, fixed overnight in 10% neutral buffered formalin at 4°C, and decalcified (Shandon’s TBD-1 Decalcifier; Thermo Scientific, Waltham, MA); then, the samples were cryoprotected by submersion in a 10–30% sucrose/PBS pH 7.4 gradient. The heads were embedded in optimal cutting temperature (OCT) compound for cryostat sectioning. For the OE sections, brain tissue was removed posterior to the OB. Tissue sections (18 μm) on glass slides were permeablized with 1% NP-40/PBS and blocked with 5% BSA. Primary antibodies for markers of olfactory sensory neurons [OMP (1:100, goat; Santa Cruz) and NeuN (1:100, mouse; Millipore)], astrocytes [GFAP (1:400, mouse; Sigma)], microglia [Iba1 (1:200, rabbit; Wako)], and oxidative stress [4-HNE (1:100, rabbit; Millipore) and 3-NT (1:100, rabbit; Abcam)] were added at 21–23°C and incubated overnight. Immunofluorescence was visualized using Alexa Fluor 488 and 594 antibodies (1:400, goat; Molecular Probes), 4’,6-diamidino-2-phenylindole (DAPI; Vector Labs), and HRP (1:400, goat; Jackson) + 3,3′-diaminobenzidine (DAB).

### Image Analysis

Images loaded onto ImageJ (Abràmoff et al. 2004) were converted into 8-bit format and were then stringently thresholded before particle and density analysis. Approximately 12 images were obtained for each mouse OB and OE per stain. For microglia, only cells larger than 20 pixels^2^ were considered for analysis. Morphology was determined based on the number of visible primary processes (30 per OB for 8 animals). Data were normalized to the pixel area of the tissue when appropriate.

### Quantitative Polymerase Chain Reaction (qPCR)

Total cellular RNA was extracted with TriReagent (Sigma) and 1-bromo-3-chloropropane (Sigma). cDNA was reverse transcribed (Superscript III kit; Invitrogen) from 2 μg RNA; qPCR was performed using appropriate primers for mouse or rat (Valuegene, San Diego, CA). For primer sequences, see Table S1. Data were normalized to GAPDH and quantified using the delta-delta-CT (cycle threshold) method.

### Statistical Analysis

Statistical analyses were performed using GraphPad Prism v.6 (GraphPad Software, Inc.). One-tailed *t*-tests were used to test single comparisons regarding inflammatory responses that have been documented in the literature (Morgan et al. 2011; Cheng et al. 2016). Two-tailed *t*-tests were used to test single comparisons regarding 4-HNE, 3-NT, nitrite, and neuronal OMP. Multiple-comparisons tests were performed using analysis of variance (ANOVA) with Tukey post-hoc tests for adjustments. Data were plotted as the mean ± SE. The threshold significance level was α = 0.05.

## Results

### Inhaled nPM Rapidly Induced Oxidative Stress in Olfactory Gateways

Cell types in brain tissue that responded to nPM *in vivo* (Levesque et al. 2011b; Morgan et al. 2011; Win-Shwe et al. 2015) were identified immunohistochemically in the OE and the OB (Figure 1A). Olfactory sensory neurons (OSNs) lined the nasal cavity, with dendrites projecting towards the external environment and away from the lamina propria (Figure 1B). Iba1-immunopositive macrophages were localized primarily in the lamina propria of the OE, with scattered presence in the OSN layer. Astrocytic GFAP was not detectable in the OSN layer but showed diffuse staining in the lamina propria without definitive astrocytic cell bodies (Figure 1B). In contrast, GFAP in the OB showed robust staining of astrocytes with classical morphology. The Iba1-positive microglia present in the OB glomerular layer are notable, as are the strongly stained OMP-positive neuronal axons (Figure 1C).

**Figure 2:**
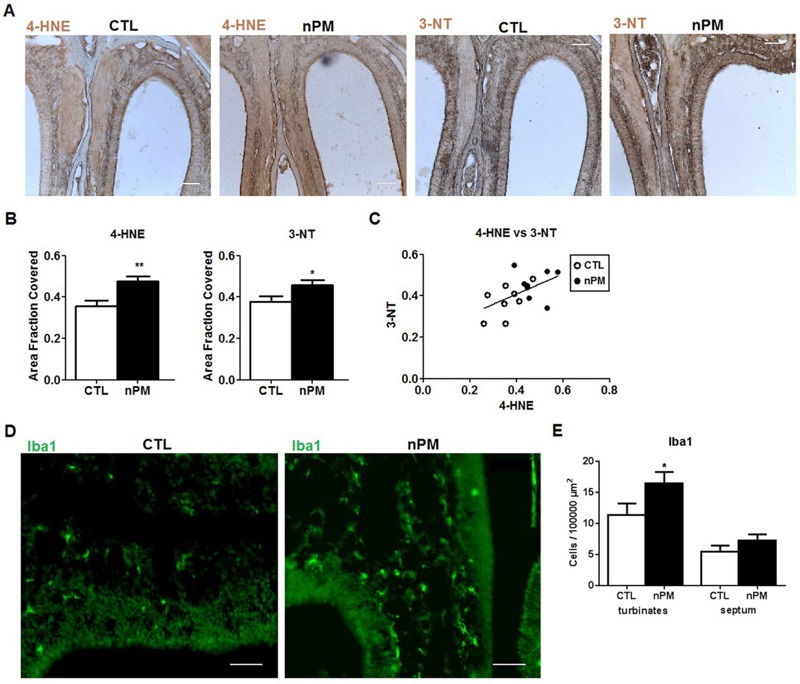
Acute *in vivo* nanoparticulate matter (nPM) exposure induced oxidative stress and inflammation in olfactory epithelium (OE): immunohistochemistry. (*A*) Representative immunostaining of 4-hydroxynonenal (4-HNE) and 3-nitrotyrosine (3-NT) in OE. (*B*) 5 hr exposure increased 4-HNE and 3-NT staining by 25% in OE versus controls (CTL) (*n* = 8 noses/group). *Scale bar* *=* 100 μm. (*C*) 4-HNE and 3-NT staining were positively correlated (*r* ^2^ = 0.27). (*D*) Iba1-positive macrophages. (*E*) Macrophage numbers increased in the OE turbinates but not in the OE septum [left: control (CTL), right: nPM]. *Scale bar* *=* 50 μm. *, *p* < 0.05; **, *p *< 0.01; *t*-test.

**Figure 3:**
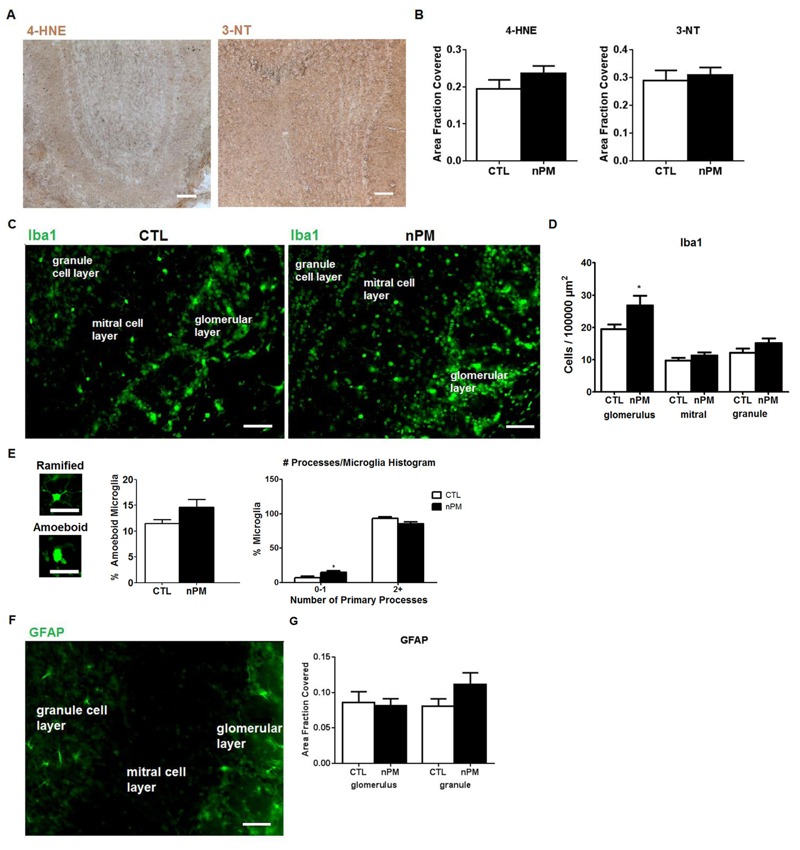
Acute *in vivo* nanoparticulate matter (nPM) exposure responses in olfactory bulb (OB): immunohistochemistry. CTL, control. (*A*) Immunostaining of 4-hydroxynonenal (4-HNE) and 3-nitrotyrosine (3-NT) in OB. (*B*) 4-HNE and 3-NT did not change significantly (*n* = 8). *Scale bar* *=* 100 μm. (*C*) Representative immunostaining of Iba1-positive macrophages. (*D*) nPM exposure increased the number of microglia in the OB glomerular layer by 30%, but no increase was observed in the mitral or granule cell layers. (*, *p *< 0.05; *t*-test). (*E*) Representative images of ramified versus amoeboid microglia. *Scale bar* *=* 25 μm. nPM doubled the percentage of microglia without multiple processes; the total percent of activated microglia did not change. [*, *p *< 0.05; two-way analysis of variance (ANOVA)]. (*F*) Immunostaining of astrocytes with glial fibrillary acidic protein (GFAP). (*G*) GFAP immunostained area by region was not altered by nPM exposure in any OB layer. *Scale bar* *=* 50 μm.

To investigate the earliest OE and OB cellular responses to nPM, adult mice were exposed to reaerosolized nPM for 5 hr and were euthanized on the next day, 18 hr later (see Figure S1). The OE showed 25% increases in the oxidative stress markers 4-HNE and 3-nitrotyrosine 3-NT (Figure 2A,B), with a positive correlation (Figure 2C). The number of Iba1-positive macrophages was increased by 30% in the OE turbinates, but those in the OE septum did not change in quantity or morphology (Figure 2D,E). In contrast to the OE, the OB did not show increased 4-HNE or 3-NT staining (Figure 3A,B). However, the number of Iba1-positive microglia in the OB glomerular layer increased by 30% without changes in the adjacent mitral or granule cell layers (Figure 3C,D). nPM doubled the number of amoeboid microglia (0–1 primary processes), which implies activation (Figure 3E). Astrocytic GFAP staining did not change in any OB layer (Figure 3F,G).

### nPM Exposure of the Olfactory Epithelium *ex Vivo*

To further investigate the initial reactions of the OE, we developed two models: *a*) *ex vivo* OE organ culture and *b*) dissociated OE primary mixed cell cultures. The OE tissue was incubated in CSF media with 12 μg/mL nPM, a concentration that induced TNFα in mixed cerebral cortical glia (Morgan et al. 2011; Cheng et al. 2016). After 2 hr of incubation, RNA responses included 30% increases in CD68, IL-1α, and TNFα (see Figure S2A).

### nPM Induced Oxidative Stress and Inflammation *in Vitro*

**Figure 4:**
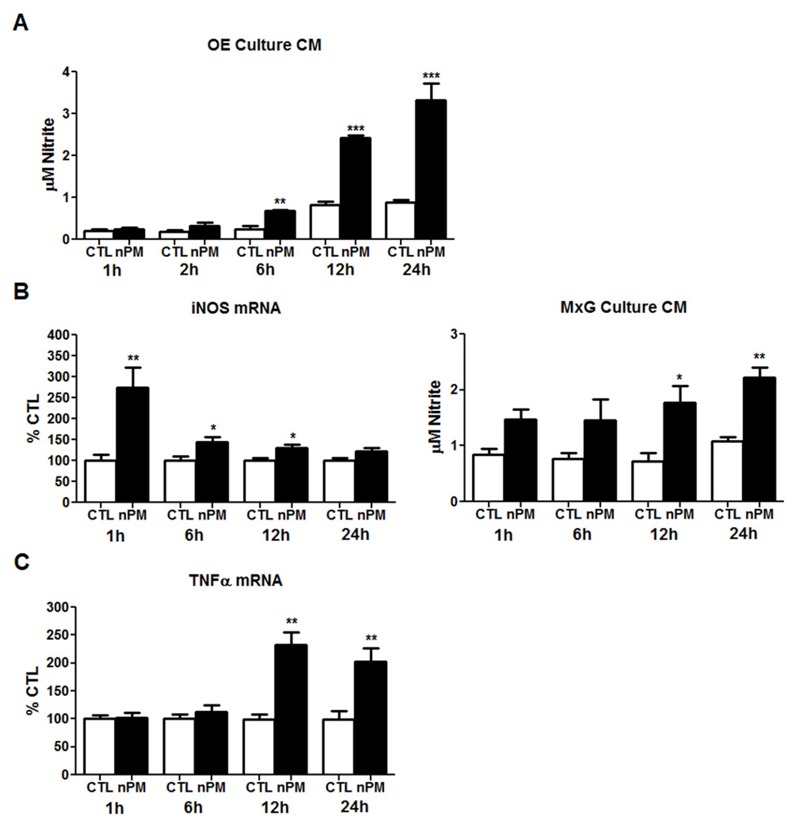
*In vitro* time-course exposure to nanoparticulate matter (nPM) induced oxidative stress and inflammation in primary cultures of rat olfactory epithelium (OE) and cerebral cortex mixed glia. CTL, control. (*A*) Conditioned media (CM) from dissociated OE cultures with 12 μg/mL nPM showed time-dependent increase of nitrite. (*B*) Cerebral cortex mixed glial (MxG) cultures with 12 μg/mL nPM transiently increased induction of induced nitric oxide synthase (iNOS) mRNA by 160%. Correspondingly, nPM doubled the CM nitrite by 12 hr. (*C*) Tumor necrosis factor-α (TNFα) mRNA was increased by 125% after 12 hr, remaining elevated at 24 hr (*n* = 6/group/time). *, *p *< 0.05; **, *p *< 0.01; ***, *p *< 0.001; *t*-test.

The induction of reactive nitrogen species was indicated by increased 3-NT (Figure 2A,B). Correspondingly, the CM from *ex vivo* OE organ culture showed a 50% increase in nitrite after 2 hr of incubation with nPM (see Figure S2B). Dissociated OE cultures also showed increased CM nitrite, continuing from 6 hr through 24 hr (Figure 4A).

Because the OE primary cell cultures included OSN cell bodies, we examined mixed cortical glia cultures, which have negligible neuronal content. During nPM exposure, nitrite in the CM also increased progressively over 24 hr (Figure 4B). Inducible nitric oxide synthase (iNOS) mRNA was rapidly, but transiently induced by 1 hr, with subsequent decrease paralleling nitrite level increases. Other nitric oxide synthases, neuronal NOS (nNOS) and endothelial NOS (eNOS), did not change with exposure to nPM or had unreliably high CT values (see Figure S3), suggesting that iNOS was the major contributor to the nitrite induction.TNFα mRNA showed slower induction, increasing after 12–24 hr (Figure 4C).

### Extended *in Vivo* nPM Exposure Induced Rapid Oxidative Stress and Inflammation in OE and OB

**Figure 5:**
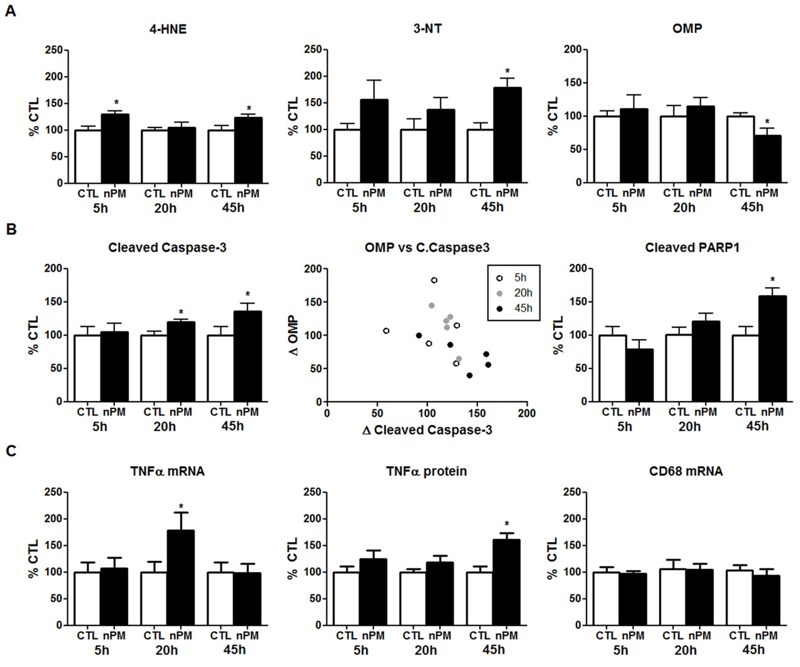
*In vivo* exposure to nanoparticulate matter (nPM) induced oxidative stress and inflammation in the olfactory epithelium (OE). (*A*) 4-Hydroxynonenal (4-HNE) adducted proteins were increased 30% after 5 hr of exposure, and these levels remained elevated at 45 hr of exposure. 3-Nitrotyrosine (3-NT) adducted proteins were increased 75% after 45 hr of exposure. Olfactory marker protein (OMP) was decreased 25% by 45 hr of exposure. (*B*) Cleaved caspase-3 showed a 20% increase by 20 hr and 45 hr of exposure. OMP varied inversely with cleaved caspase-3 (Spearman correlation, *r* = –0.61). Each data point represents the percent change versus controls (CTL). Cleaved poly(ADP-ribose) polymerase 1 (PARP1), an indirect product of cleaved caspase-3 activity, was increased 60% after 45 hr of exposure. (*C*) Tumor necrosis factor-α (TNFα) mRNA transiently increased 75% in OE after 20 hr of cumulative nPM exposure versus controls. TNFα protein increased 60% at 45 hr. CD68 did not change (*n* = 6 mice/group/time). *, *p* < 0.05; *t*-test.

We extended the *in vivo* time course of nPM response to total exposures of ≤ 45 hr over 3 weeks (see Figure S1). Corresponding to the immunohistochemistry of the OE (Figure 2), Western blots of the OE showed a 30% increase in 4-HNE that persisted from 5 to 45 hr of total nPM exposure (Figure 5A). In addition, 3-NT showed a trend for increase at 5 hr and was significantly increased by 75% at 45 hr (Figure 5A). OMP, expressed only by mature OSNs, was reduced by 25% at 45 hr of nPM exposure (Figure 5A). The decrease in OMP varied inversely with levels of cleaved caspase-3, a marker of apoptosis (Figure 5B). As an indirect measurement of cleaved caspase-3 activity, poly(ADP-ribose) polymerase 1 (PARP1) also increased at 45 hr in the OE (Figure 5B). TNFα responded more slowly: the only significant changes were in TNFα mRNA (+75%) at 20 hr and in TNFα protein (+60%) at 45 hr (Figure 5C). IL-1α mRNA fluctuated, with possible transient increase at 20 hr (not shown). There were no responses of the microglial marker CD68 (Figure 5C).

The OB showed similarly modest inflammatory and oxidative stress responses to extended nPM exposure (see Figure S4). TNFα mRNA, but not protein, showed a transient increase at 20 hr of exposure, whereas TNFα protein was only increased at 45 hr. CD68 mRNA was more consistently increased at 20 hr and 45 hr (see Figure S4A). Unlike in the OE, 4-HNE and 3-NT increased in the OB only at 45 hr (see Figure S4B). OMP in the OB did not respond at any time (not shown).

### Downstream Responses of the Cerebral Cortex and Cerebellum

**Figure 6:**
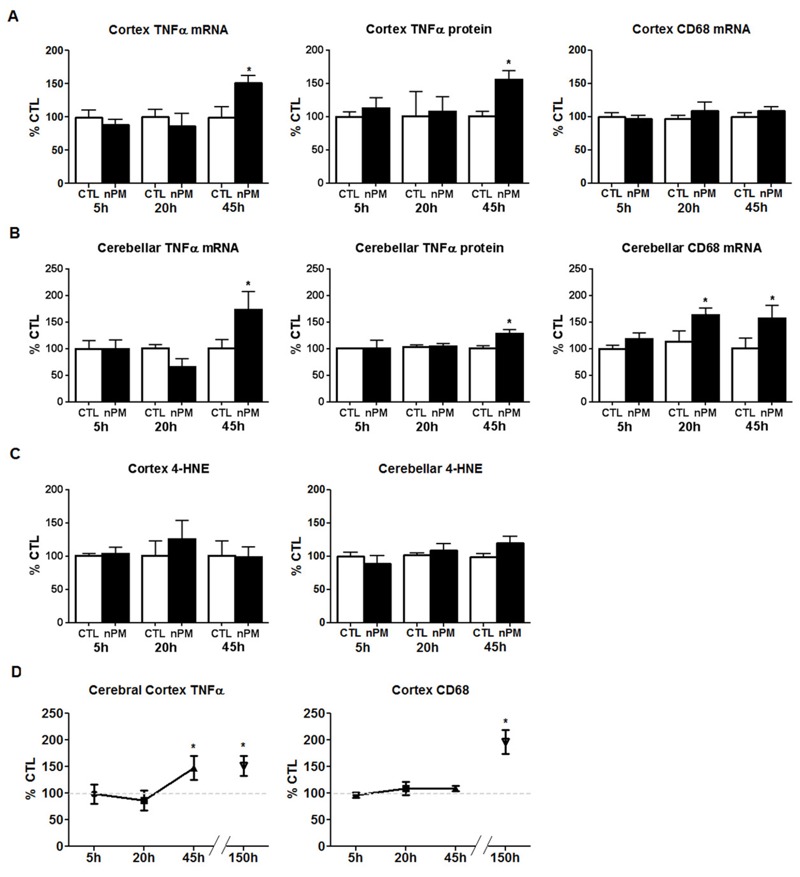
Extended *in vivo* exposure to nanoparticulate matter (nPM) induced tumor necrosis factor-α (TNFα) in the cerebral cortex and cerebellum. (*A*) TNFα mRNA increased 50% in cortex after 45 hr of cumulative nPM exposure versus controls (CTL) (*n* = 6 mice/group/time). TNFα protein increased 50% after 45 hr. CD68 mRNA did not change. (*B*) Cerebellar TNFα mRNA increased 70% after 45 hr; TNFα protein increased by 30% after 45 hr. Cerebellar CD68 mRNA increased 50% at 20 hr and 45 hr. (*C*) 4-Hydroxynonenal (4-HNE) did not change in cerebral cortex or cerebellum. (*D*) Data from the present study are graphed with prior findings from 150 hr nPM exposure, which increased TNFα and CD68 by 50% and 90% respectively (Morgan et al. 2011). *, *p* < 0.05; *t*-test.

We assayed the cerebral cortex and the cerebellum for comparison with the OE and the OB. TNFα mRNA increased 50% in cortex and 70% in cerebellum at 45 hr (Figure 6A,B,D). In parallel to mRNA, TNFα protein increased 50% in cortex and 30% in cerebellum at 45 hr (Figure 6A,B). Cerebellar CD68 mRNA was increased 50% at 20 hr and 45 hr, but cortex CD68 did not respond (Figure 6A,B). Moreover, 3-NT (not shown) and 4-HNE (Figure 6C) did not change in either region. For comparison with 150 hr total exposure over 10 weeks (Morgan et al. 2011), Figure 6D shows combined data for cerebral cortex CD68 and TNFα mRNA.

## Discussion

To our knowledge, these are the first data on the acute cell responses of the nasal olfactory epithelium (OE) to air pollution PM and the first time course of spatial responses to any air pollutant from nose to brain regions. In these experiments, rodent neural tissues were exposed to nPM, a nanosized subfraction of PM_2.5_ that was enriched in water-soluble organic compounds. The *in vivo*, *ex vivo*, and *in vitro* models of the OE showed a rapid increase in oxidative stress by 24 hr of nPM exposure, with increased tissue levels of 4-HNE, 3-NT, and TNFα (Figures 2, 4, and 5; see also Figure S2).

We anticipated that the OB would also show rapid responses because it has been previously shown that olfactory neurons of the OE axonally transmitted nanoscale gold PM into the OB (De Lorenzo 1970) and that inhaled nanoscale PM of ^14^C-graphite and of intranasal Mn were rapidly accumulated in the OB (Elder et al 2006; Oberdörster et al. 2004). However, the OB had small increases of 4-HNE and 3-NT and slow responses of TNFα until late in the series of exposures, as did cerebral cortex and cerebellum (Figure 6; see also Figure S4). Only at 45 hr of total exposure to nPM over 3 weeks did cerebral cortex TNFα mRNA approach the levels measured at 150 hr of nPM exposure over 10 weeks in our initial study (Morgan et al. 2011). These delayed responses suggest contributions from systemic responses to nPM that may interact with the direct olfactory nerve pathway from nose to brain. We also consider a neuronal degenerative response of the OE that differed from downstream brain regions.

The OSNs that line the OE are the only neurons in the respiratory tract that are directly exposed to the external environment and are, therefore, the first neuronal responders to inhaled air pollutants. Early increases of 4-HNE and 3-NT after a 5-hr exposure to nPM were histochemically localized to the nasal epithelial mucosa, concurrent with increased numbers of macrophages (Iba1-positive) in the turbinate zone (Figure 2). However, TNFα increases were delayed in the OE until 20–45 hr, as was a neuronal degenerative response, as shown in the 25% reduction of OMP with a reciprocal increase of cleaved caspase-3 (Figure 5).

Responses in the OB were more modest and delayed than those in the OE. Although 4-HNE and 3-NT had increased in the OB by 45 hr (see Figure S4), we did not detect any change in neuronal OMP, unlike in the OE. Furthermore, neither the downstream cerebral cortex nor the cerebellum showed increased 4-HNE or 3-NT at any time, and TNFα was only increased at 45 hr (Figure 6). We anticipate that longer exposure to nPM would increase 3-NT in the brain, as observed for 80 hr of exposure to diesel exhaust, which increased whole-brain 3-NT by > 100% (Levesque et al. 2011b). Because the cerebral cortex and the cerebellum are at least two synapses from the OSN (Kronenbuerger et al. 2010), it is notable that their TNFα induction was similar in size to TNFα increases in the OB and OE. The relatively larger size of these nasally distant brain regions raises an important question: one might expect that transynaptically transported nPM would be diluted in some proportion to the brain mass, from the OB (25 mg wet weight) to the cerebral cortex (200 mg) and the more remote cerebellum (70 mg), yet the TNFα induction was similar. Could systemic mechanisms be involved?

Several lines of evidence support the role of systemic import of particulate material or of proinflammatory factors. Oberdörster et al. (2004) reported that a single exposure to inhaled nanoscale ^14^C-graphite caused brain levels of ^14^C that were as high or higher in cerebellum than those in the OB after 5 days. These authors discussed possible “…translocation across the blood–brain barrier in certain regions” (Oberdörster et al. 2004). A blood-borne source of the persisting ^14^C elevations in cerebellum and cerebrum would be consistent with the large residual ^14^C pool in the lung. Further evidence for a lung-to-brain axis in air pollution comes from brain responses to intratracheal instillation of a PM_10_ air pollutant fraction, which increased induction of the oxidatively sensitive gene heme oxygenase 1 (HO-1) by > 100% in both whole brain and lung (Farina et al. 2013). Systemic transmission of particles or inflammatory factors is also consistent with effects on fetal brain from intratracheal diesel exhaust particles (DEP) (Bolton et al. 2014). Moreover, inhaled vehicular emissions increased the permeability of the blood-brain barrier (BBB) in mice, and serum from pollution-exposed mice increased BBB permeability in an *in vitro* model (Oppenheim et al. 2013) and altered vasorelaxation with CD36 dependence (Robertson et al. 2013). Lastly, we note that increased circulating cytokines from respiratory tract inflammation can cross the BBB and evoke neuroinflammatory responses (Erickson et al. 2012; Peters et al. 2006). Thus, systemic effects of air pollution warrant further study in conjunction with the established direct nose–brain pathway. We anticipate complex transitions of pathway-specific mechanisms during prolonged exposures.

The induction of TNFα, 4-HNE, and 3-NT implicate oxidative and inflammatory mechanisms during *in vivo* exposure. The rapid increase in 4-HNE and 3-NT in OE was associated with nitrosative stress with several *in vitro* models. *Ex vivo* intact OE was incubated with nPM under conditions based on prior studies of hippocampal slices, in which nPM increased nitric oxide (NO) and *S*-nitrosylation (Davis et al. 2013). The *ex vivo* and primary OE cultures showed increased nitrite in response to nPM (Figure 4; see also Figure S2). Mixed glia from cerebral cortex also responded to nPM with induced nitrite and iNOS. The *ex vivo* OE also showed induction of cytokines (IL-1α, TNFα) and macrophage activation (CD68). The neonatal rat OE had similar *ex vivo* responses (Cheng et al. 2016). These changes parallel the *in vivo* rapid inflammatory responses to nPM inhalation, which include increased Iba1-positive macrophages in the OE turbinate layer and in the OB glomerular layer (Figures 2 and 3).

The inflammatory changes observed in the OE and the brain may be propagated by macrophage/microglial activation in response to oxidative stress induced by nPM. Macrophage scavenger receptors, including CD36, can be activated by 4-HNE (Ishii et al. 2004; Stewart et al. 2010). Additionally, adducts of HNE modified by glutathione may activate NF-κB (Ramana et al. 2006) as well as induce TNFα. These oxidative markers, 4-HNE and 3-NT, are implicated in the pathogenesis of Alzheimer disease (AD) and other neurodegenerative disorders (Butterfield et al. 2007; Dalleau et al. 2013; Shringarpure et al. 2000).

There are notable variations of OB responses between exposure models (Gerlofs-Nijland et al. 2010; Guerra et al. 2013; Levesque et al. 2011a). The lack of, or attenuated, OB responses in the longer exposures with baseline return of TNFα mRNA by 45 hr observed in the present study suggests compensatory OB mechanisms. This finding warrants further attention: Ong et al. (2016) showed that a single 4-hr exposure over 1 day to 0.5 ppm ozone, a gaseous pollutant absent from nPM, transiently increased induction of TNFα mRNA by 70% in the nasal mucosa, with a return to control levels by day 4 of exposure (Ong et al. 2016 ; Wagner et al. 2002). Moreover, there was rapid infiltration of neutrophils into the nasal mucosa within 2 hr after the initial exposure. In our model, the lack of TNFα, IL-1β, and CD68 mRNA responses in the OE and the OB after 1 day of nPM inhalation suggests that ozone-mediated toxicity in nasal mucosa occurs more rapidly through different mechanisms.

Neurodegenerative changes in olfactory neurons arose much later in the OE, indicated by a reduction in levels of the OSN-specific protein OMP with inverse proportion to the apoptosis marker cleaved caspase-3 (Figure 5A,B). The induction of TNFα in the OE may interfere with OSN regeneration (Turner et al. 2010). Because OSNs are exposed to inhaled environmental toxins, they are continuously regenerated in conjunction with apoptosis to maintain functionality (Holcomb et al. 1996). Moreover, extended chronic exposure to ultrafine PM also induced apoptosis (Win-Shwe and Fujimaki 2011). These observations are consistent with those from studies performed on domestic dogs from a highly polluted city, which showed disrupted OSN and sustentacular cell layers, with an overall thinning of the OE (Calderón-Garcidueñas et al. 2003). Relevant to humans, late-onset olfactory dysfunction is a risk factor for AD (Arnold et al. 2010; Devanand et al. 2000).

*In vitro* models of the OE may help identify specific air pollution component cytotoxic activities. For example, we do not know the role of free radicals that persist in nPM for at least 30 days after initial collection and subsequent reaerosolization (Morgan et al. 2011). Further fractionation of nPM could also resolve the activities of particular water-insoluble organic compounds.

## Conclusions

These data support the hypothesis that inhaled nPM rapidly causes oxidative stress in the OE, with delayed inflammatory and neurodegenerative responses that include increased apoptosis of olfactory neurons. Although the OB receives direct input from olfactory neurons, its inflammatory and oxidative stress responses were delayed. The cerebral cortex and cerebellum also responded with a slower increase of TNFα, but these regions did not show increases in nitrosylated proteins or oxidized lipids at any time. These delayed brain responses suggest that inhaled nPM in the OE and the OB contribute to neurodegenerative effects of air pollution particulates. Systemic factors also merit further consideration in brain responses to air pollution. We hypothesize that the brain-wide increases in TNFα and other cytokines occurring with exposure to air pollution contribute to the cognitive deficits that have been epidemiologically associated with air pollutants. TNFα has well-defined links to glutamatergic functions in the hippocampus and cerebral cortex (Beattie et al. 2002; Santello and Volterra 2012) that are critical to learning and memory.

## Supplemental Material

(330 KB) PDF
