# Supplementary material for: Nanoscale Particulate Matter from Urban Traffic Rapidly Induces Oxidative Stress and Inflammation in Olfactory Epithelium with Concomitant Effects on Brain
Source: Environ Health Perspect. 2016 May 17;124(10):1537–46. doi: 10.1289/EHP134 (PMC5047762; doi:10.1289/EHP134)
Supplement: (330 KB) PDF [file EHP134.s001.acco.pdf]

Environ Health Perspect
DOI: 10.1289/EHP134

Note to readers with disabilities: *EHP* strives to ensure that all journal content is accessible to all readers. However, some figures and Supplemental Material published in *EHP* articles may not conform to 508 standards due to the complexity of the information being presented. If you need assistance accessing journal content, please contact ehp508@niehs.nih.gov. Our staff will work with you to assess and meet your accessibility needs within 3 working days.

Supplemental Material

Nano-Scale Particulate Matter from Urban Traffic Rapidly Induces Oxidative Stress and Inflammation in Olfactory Epithelium with Concomitant Effects on Brain

Hank Cheng, Arian Saffari, Constantinos Sioutas, Henry J. Forman, Todd E. Morgan, and Caleb E. Finch

Table of Contents

Figure S1. Time course of nPM exposure. Mice were exposed to re-aerosolized nPM (343 $\mu g/m^3$) for 5 h/day, 3 d/week for a total of 5, 20, and 45 cumulative hours. Tissues were collected 18 h after the last exposure.

Figure S2. Ex vivo exposure to nPM rapidly induced inflammatory responses in OE. (A) Ex vivo treatment of OE for 2 h with 12 µg/ml nPM induced TNFα, IL-1α, and CD68 mRNAs by 30% (n = 8 noses/group); PCR CT range: TNFα 28-30, IL-1α 28-30, and CD68 24-26. (B) Nitrite in the ex vivo OE conditioned media (CM) increased 50%. (*; $p<0.05$; **; $p<0.01$; t-test)

Figure S3. In vitro time course exposure to nPM did not induce nNOS or eNOS in cerebral cortex mixed glia. (A) nPM did not affect nNOS expression vs controls at any time except at 1 h, where nNOS was decreased 30%. (B) eNOS CT values were above reliable quantification (CT >30) at all times. Samples that did not yield melting curves were removed (n=6/group/time). (*; $p<0.05$; t-test).

Figure S4. nPM in vivo exposure induced oxidative stress and inflammation in the OB. (A) TNFα mRNA transiently increased by 90% in OB after 20 h of cumulative nPM exposure vs. controls (n = 6 mice/group/time). TNFα protein was increased 60% after 45 h of total exposure. CD68 mRNA increased by 25% by 20 h and 45 h. (B) 4-HNE and 3-NT increased 50% by 45 h. (*; $p<0.05$; t-test).

Table S1. Primer sequences.

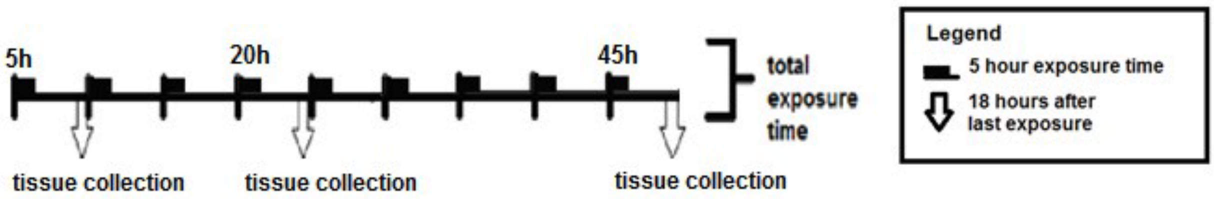

Figure S1. Time course of nPM exposure. Mice were exposed to re-aerosolized nPM (343 μg/m^3) for 5 h/day, 3 d/week for a total of 5, 20, and 45 cumulative hours. Tissues were collected 18 h after the last exposure.

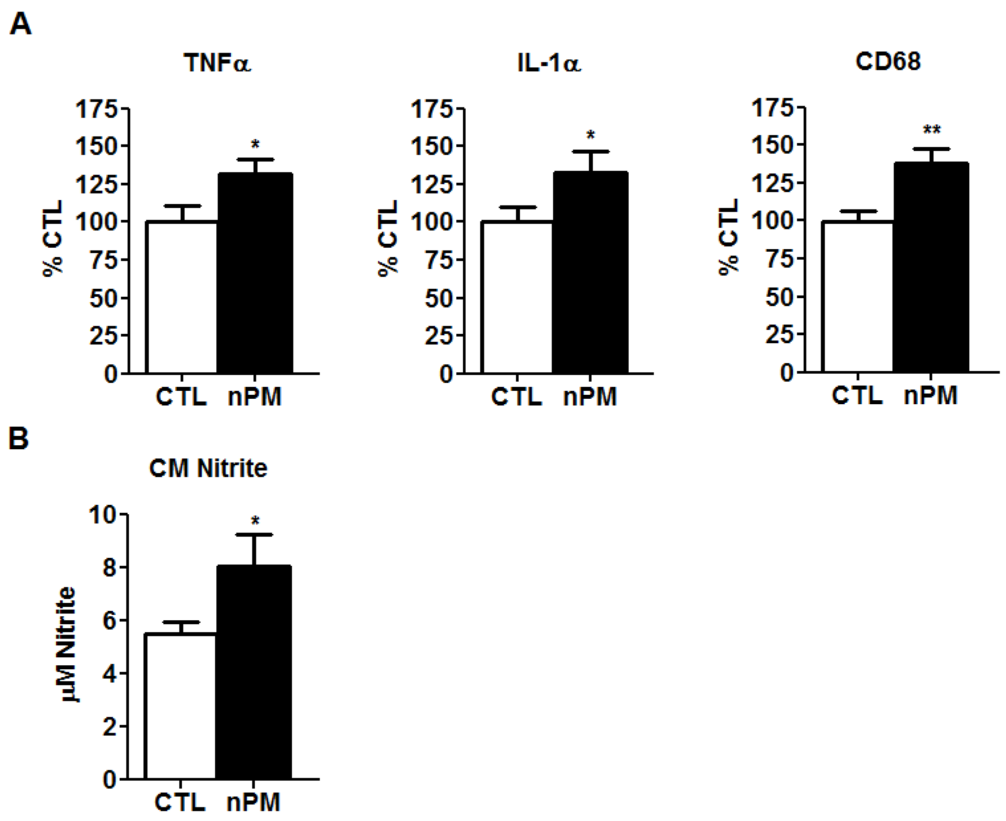

Figure S2. Ex vivo exposure to nPM rapidly induced inflammatory responses in OE. (A) Ex vivo treatment of OE for 2 h with 12 μg/ml nPM induced TNFα, IL-1α, and CD68 mRNAs by 30% (n = 8 noses/group); PCR CT range: TNFα 28-30, IL-1α 28-30, and CD68 24-26. (B) Nitrite in the ex vivo OE conditioned media (CM) increased 50%. (*; $p<0.05$; **; $p<0.01$; t-test)

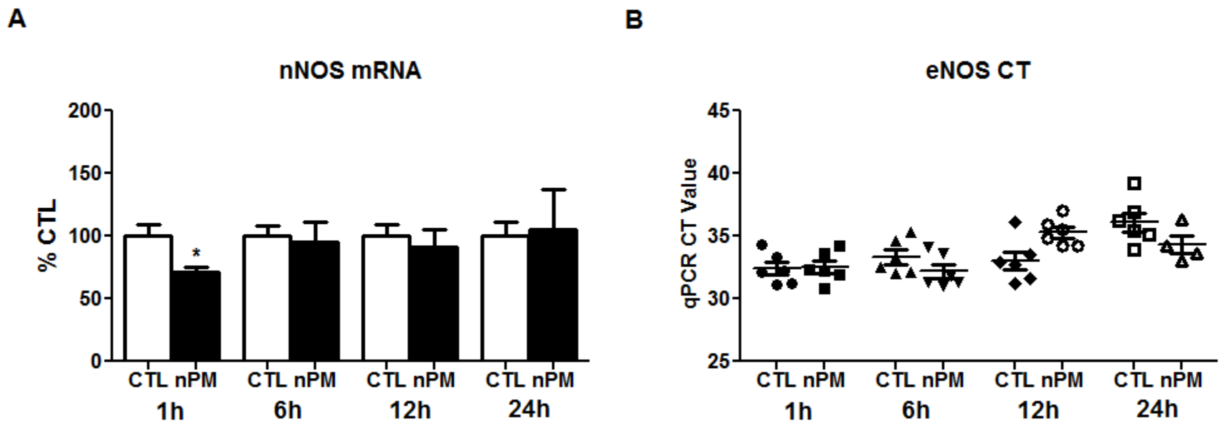

Figure S3. In vitro time course exposure to nPM did not induce nNOS or eNOS in cerebral cortex mixed glia. (A) nPM did not affect nNOS expression vs controls at any time except at 1 h, where nNOS was decreased 30%. (B) eNOS CT values were above reliable quantification (CT >30) at all times. Samples that did not yield melting curves were removed (n=6/group/time). (*; $p<0.05$; t-test).

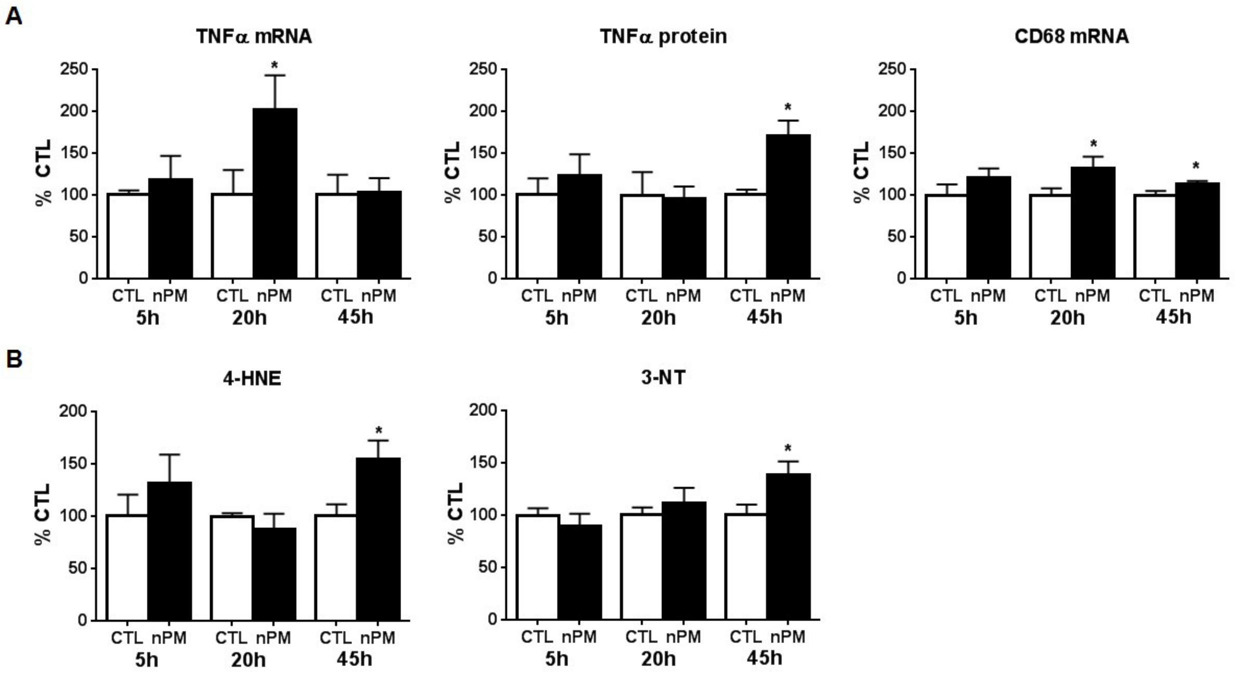

Figure S4. nPM in vivo exposure induced oxidative stress and inflammation in the OB. (A) TNFα mRNA transiently increased by 90% in OB after 20 h of cumulative nPM exposure vs. controls (n = 6 mice/group/time). TNFα protein was increased 60% after 45 h of total exposure. CD68 mRNA increased by 25% by 20 h and 45 h. (B) 4-HNE and 3-NT increased 50% by 45 h. (*; $p<0.05$; t-test).

Gene	Forward (5' – 3')	Reverse (5' – 3')
r TNF α	CGTCAGCCGATTTGCTATCT	CGGACTCCGCAAAGTCTAAG
r IL-1 α	TCGGGAGGAGACGACTCTAA	GTGCACCCGACTTTGTTCTT
r CD68	TTCTGCTGTGGAAATGCAAG	AGAGGGGCTGGTAGGTTGAT
r iNOS	CATTGGAAGTGAAGCGTTTCG	CAGCTGGGCTGTACAAACCTT
r nNOS	GCCAAGACCCTGTGTGAGAT	AGCCGAATTTCTCCCCGTTC
r eNOS	GCAGTGGAAATTAACGTGGCT	GGCCTTCTGCTCATTTTCCAAG
r GAPDH	AGACAGCCGCATCTTCTTGT	CTTGCCGTGGGTAGAGTCAT
m TNF α	CGTCAGCCGATTTGCTATCT	CGGACTCCGCAAAGTCTAAG
m IL-1 α	TCGGGAGGAGACGACTCTAA	GTGCACCCGACTTTGTTCTT
m CD68	CCAATTCAGGGTGGAAGAAA	CTCGGGCTCTGATGTAGGTC
m GAPDH	CCAATGTGTCCGTCGTGGATCT	GTTGAAGTCGCAGGAGACAACC

m denotes mouse; r, rat

Table S1. Primer sequences.
